# New developments in diagnostics and treatment of adult-onset focal dystonia

**DOI:** 10.1097/WCO.0000000000001165

**Published:** 2023-05-31

**Authors:** Liesanne M. Centen, Martje E. van Egmond, Marina A.J. Tijssen

**Affiliations:** a Department of Neurology; b Expertise Center Movement Disorders Groningen, University of Groningen, University Medical Centre Groningen, Groningen, the Netherlands

**Keywords:** adult-onset, diagnosis, focal dystonia, treatment

## Abstract

The aim of this review is to showcase the recent developments in the field of diagnosis and treatment of adult-onset focal dystonia.

**Recent findings:**

Accurate phenotyping of focal dystonia is essential in the process of finding an underlying cause, including acquired, genetic, and idiopathic causes. Motor symptoms as well as the associated nonmotor symptoms and their detrimental impact on quality of life have received increased interest over the last years. The diagnostic process is complicated by the steadily increasing numbers of newly discovered genes associated with dystonia. Recent efforts have been aimed at further developing recommendations and algorithms to aid in diagnosis and in navigating the use of diagnostic tools. In terms of treatment, research on DBS is advancing towards a better understanding of the most effective stimulation locations within the globus pallidus. Moreover, with the introduction of the LFP-recording devices, the search continues for an accurate electrophysiological biomarker for dystonia.

**Summary:**

Accurate phenotyping and (sub)classification of patients with dystonia is important for improving diagnosis, subsequent treatment effect and population-based study outcomes in research. Medical practitioners should be attentive to the presence of nonmotor symptoms in dystonia.

## INTRODUCTION

Dystonia is a hyperkinetic movement disorder characterized by sustained muscle contractions causing abnormal movements and postures or both [1]. The phrase ‘dystonia’ was first coined by German neurologist Herman Oppenheim in 1911, and its definition has since undergone many modifications [2]. The most common form is adult-onset focal dystonia, in which usually either the neck, eyelids, voice, mouth, or upper limb are affected. These are isolated forms of dystonia, indicating that the clinical picture does not involve other movement disorders or neurological phenomena, apart from accompaniment by tremor [1]. In most of these cases, the dystonia is confined to one body region; however, spread to a neighboring region is possible [3]. Due to its heterogenous presentation, diagnosing adult-onset focal dystonia accurately remains a challenge. The absence of widely accepted diagnostic criteria for the different subtypes complicates this matter.

In addition to the visible motor symptoms, an increasing body of evidence shows that nonmotor symptoms (NMS) are commonly associated with adult-onset focal dystonia. Recent studies are calling for a more holistic approach in the treatment of dystonia, as NMS significantly impact quality of life. 

**Box 1:**
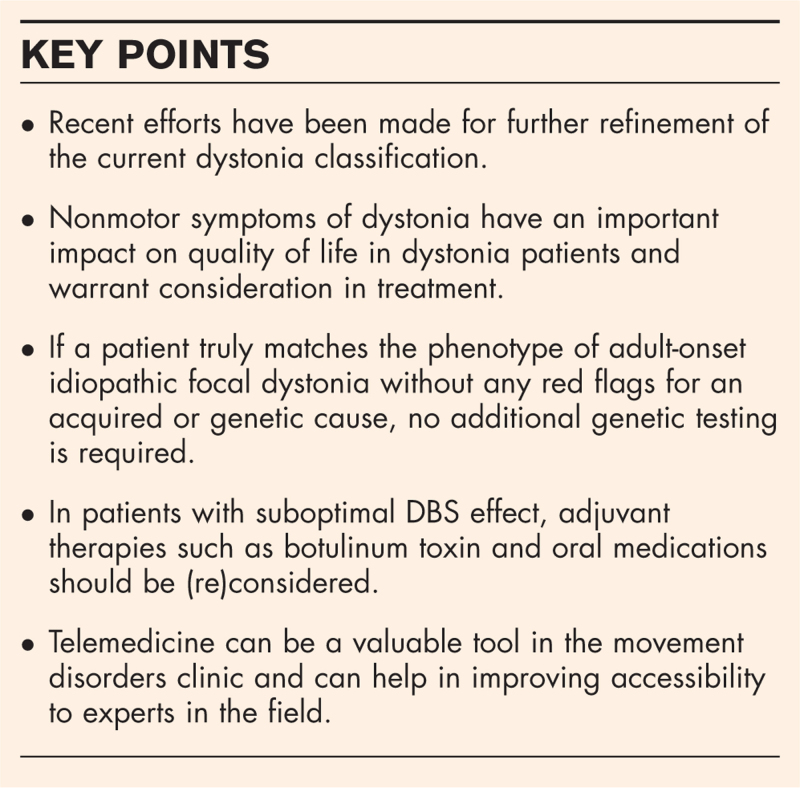
no caption available

## CLINICAL FEATURES AND RECOMMENDATIONS FOR DIAGNOSIS

Dystonia is a heterogenous disorder in its clinical presentation and can be difficult to diagnose. Over time, several propositions for a classification of dystonia have been made. Nowadays, the 2013 consensus statement by the Movement Disorder Society (MDS) regarding the classification for dystonia has been universally implemented effectively. Nonetheless, as concluded in previous studies, these guidelines allow for some leeway in clinical judgement, sometimes leading to varying opinions or conclusions [4]. Acknowledging this issue, several efforts have been made recently to provide further recommendations for diagnosis of blepharospasm (BSP) and idiopathic cervical dystonia [5^▪^,6^▪^,^7^]. In cervical dystonia it is shown that establishing the difference in diagnosis between segmental or focal dystonia can be challenging. In a recent cohort of 1258 patients diagnosed with focal cervical dystonia was found that in 28.2% of cases, dystonia outside of the neck region was found. With the most common region being the shoulder (13.4%), followed by dystonia of the hand (2.6%), upper face (2.1%), upper arm (1.2%), larynx (1.1%), lower face (0.7%), trunk (0.7%) and jaw and tongue (0.3%). This study provides a recommendation for cervical dystonia, in which is proposed to reserve the diagnosis of focal cervical dystonia for those patients in which only muscles that move the neck are involved [6^▪^] (Table 1). Recently, the MDS has published a consensus statement regarding the classification of idiopathic cervical dystonia, differentiating between the two levels ‘definite’ and ‘probably’ idiopathic cervical dystonia [8^▪▪^]. Supportive criteria and exclusion criteria for cervical dystonia diagnosis were identified (Tables 2 and 3). Additionally, required criteria are also provided for diagnosis of genetic and acquired cervical dystonia [8^▪▪^].

**Table 1:** Recommendations for diagnosis of cervical dystonia according to body regions affected

Diagnosis	Body regions involved
Focal cervical dystonia	Neck only Neck plus shoulder Neck plus platysma
Segmental dystonia with neck involvement	Neck plus shoulder and upper arm Neck plus shoulder and whole arm/hand Neck plus jaw/tongue Neck plus lower face Neck plus larynx Neck plus trunk
Multifocal dystonia with neck involvement	Neck plus hand (excluding upper arm) Neck plus upper face (not including lower face, jaw, or tongue) Neck plus lower limb
Generalized dystonia with neck involvement	Neck plus trunk plus at least one other body region

Source: Kilic-Berkmen *et al.*[6^▪^].

**Table 2:** Supportive criteria for diagnosis of idiopathic cervical dystonia

1. At least two abnormal cervical positions are recognized as part of the individual phenomenology
2. Dystonic movements are patterned, having consistent directionality and predictability
3. Effective alleviating maneuvers are observed
4. Pain is improved by botulinum neurotoxin injections in overactive muscles
5. If there is dystonic head tremor, a null point is detectable
6. Age at onset is typical (between 30 and 70 years)

Source: Albanese *et al.*[8^▪▪^].

**Table 3:** Exclusion criteria for diagnosis of idiopathic cervical dystonia

1. Occurrence of additional movement disorders, ataxia, unexplained pyramidal tract signs, other neurological or systemic features
2. Unexplained cognitive impairment
3. Dystonia is paroxysmal
4. Dystonia is generalized
5. Condition that mimics idiopathic cervical dystonia

Source: Albanese *et al*. [8^▪▪^].

Similar in BSP is the lack of universal diagnostic criteria. Based on their earlier work on BSP, Defazio *et al.* have identified four clinical items: stereotyped, bilateral, and synchronous orbicularis oculi spasms, presence of a sensory trick, increased blinking frequency, and inability to suppress the spasms. Accuracy of these diagnostic items in a large population has been validated, and their use in practice is endorsed in multiethnic, multicenter cohorts [5^▪^]. The development and use of universal clinical guidelines is important as it improves consistency and accuracy of diagnosis.

## NONMOTOR SYMPTOMS

Recent studies show that the disease burden of dystonia stretches far beyond the visible motor symptoms, as NMS greatly impact quality of life [9,10]. It is hypothesized that these symptoms are a fundamental part of the disease, as they can predate the onset of motor symptoms by several years [11]. The presence of NMS is reported across the entire spectrum of adult-onset focal dystonia [12–15]. There is evidence in cervical dystonia and BSP that different subtypes exist with varying degrees of motor severity and presence of NMS [12,15]. The subject of NMS has been extensively studied in cervical dystonia. In a recent study conducting the EQ-5D-5L among patients with cervical dystonia, particularly depression, anxiety, pain, and sleep quality were important factors in health-related quality of life [16], supporting previous studies in cervical dystonia, and also in other types of adult-onset focal dystonia [13,17]. Thus far, there has been little consideration of treatment specifically aimed at improving NMS. It appears that botulinum toxin treatment (BoNT) can have a beneficial effect on some of the NMS, but reported outcomes are contradictory [18–20]. For DBS reporting of NMS, especially in adult-onset focal dystonia, is scarce. A review based on studies with limited cohort sizes showed positive effects on pain [21], and a prospective study in generalized dystonia demonstrated improvement in a wide array of NMS [22^▪^]. Nonetheless, there is a promising development with several recent publications stressing the importance of NMS in the treatment of patients with dystonia, advocating for a more holistic approach [23–25].

## CAUSE OF ADULT-ONSET DYSTONIA

**FIGURE 1:**
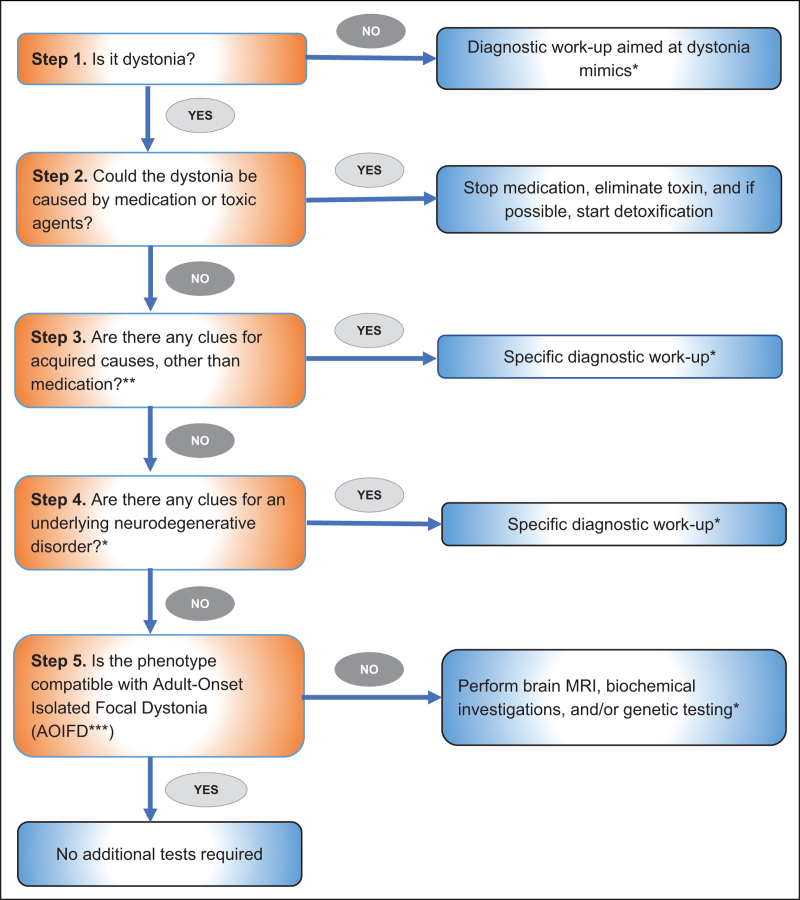
A novel five-step diagnostic algorithm for adult-onset dystonia. ^∗^For tables on specific diagnostic workup recommendations, see the original article [26^▪▪^]. ^∗∗^The authors recommend TSH (thyroid-stimulating hormone) screening in all patients. ^∗∗∗^For table on the clinical characteristics of adult-onset isolated focal dystonia (AOIFD), see the original article [26^▪▪^]. Source: van Egmond *et al*. [26^▪▪^].

A clinical diagnosis of adult-onset dystonia is generally based on phenomenology. Most cases are of idiopathic cause, requiring no further testing to establish a diagnosis. However, for a small percentage of patients, the dystonia is caused by a treatable underlying acquired or a genetic disease (e.g. medication-induced dystonia, Wilson's disease, dopa-responsive dystonia), thus prompt and accurate diagnosis is crucial in these cases. On top of that, the increasing numbers of newly discovered genes associated with adult-onset dystonia does raise the question to clinicians when further testing is justified. In a recent study, a diagnostic algorithm was designed based on extensive literature review and expert opinion to aid in the decision-making process in these cases [26^▪▪^] (Fig. 1). The algorithm consists of five steps, with the first four steps aimed at accurate phenotyping of dystonia and recognizing acquired or neurodegenerative causes. The fifth step is aimed at determining whether genetic testing is indicated. Out of all patients that were included in the literature review (*n* = 17 127), merely 1.05% had an underlying genetic mutation, and for only 0.01% of patients (*n* = 2), the dystonia was proven to be caused by a treatable genetic disorder. As a result, the authors conclude that genetic testing is currently not necessary if red flags for a genetic cause are lacking, and the phenotype of the patient truly matches that of adult-onset idiopathic focal dystonia.

## NOMENCLATURE OF GENETIC DYSTONIA

With the vastly expanding number of genes discovered associated with dystonia and other movement disorders comes the challenge to keep the nomenclature for these disorders as structured as possible. Recently, an update has been performed by the MDS Task Force for the Nomenclature of Genetic Movement Disorders on the most recently discovered genes for several movement disorders. In total, 21 genes associated with dystonia have been updated with a prefix [27^▪^]. Of importance for adult-onset dystonia is the *DYT-ANO3* gene, associated with cranial–cervical dystonia with variable age of onset and usually combined with tremor and myoclonus.

## PHARMACOLOGICAL TREATMENT

BoNT persists as the mainstay of treatment for focal dystonia. Its efficacy has been proven countless times in many studies and trials, with long-term follow-ups also showing continued safety and efficacy in up to over two decades of treatment [28–31]. There are several different formulations of botulinum toxin available nowadays. A recent review shows that efficacy of the different types is largely comparable [32^▪^]. The review also concludes that, other than for antiepileptics, for most nontoxin pharmacotherapeutics the evidence for effectiveness is based on limited research. Promising experimental pharmacotherapeutics mentioned are dipraglurant, which modulates the metabolic glutamate receptor type 5 (mGluR5), which is involved in the development of dystonia in DYT1 rodent models, and sodium oxybate, studied in alcohol-responsive spasmodic dysphonia patients [32^▪^].

## DEEP BRAIN STIMULATION

Deep brain stimulation (DBS) is an effective advanced treatment option for medication refractory dystonia. To date, the Globus Pallidus interna (GPi) has been the most regularly targeted area in dystonia. Its efficacy in focal, segmental, and generalized dystonia has been studied extensively in both short-term and long-term studies [33–39].

An alternative emerging target is the subthalamic nucleus (STN). Two studies published in the last year both demonstrate a remarkable long-term improvement in CD symptoms during a follow-up of up to 2, and up to 15 years [40^▪^,^41^]. In terms of efficacy compared with the GPi as target, a previously conducted meta-analysis discovered no difference between the two targets. Important to note is that each comes with its own stimulation-related adverse effect profile [42]. Both targets are associated with side effects such as paresthesias, dysarthria, dysphagia, balance problems, and gait disturbances [42], but mood symptoms (dysphoria, depression, and anxiety), weight gain, and dyskinesias are linked to the STN [43], while bradykinesia and coordination problems are linked to the GPi [44,45].

In general, the response of dystonia patients to DBS remains variable and often difficult to predict. Around 10–25% of patients undergoing DBS can be regarded as nonresponders and experience less than 25% improvement [35]. Technical factors such as electrode location play an important role in this but do explain only part of the reduced response [46]. Other factors associated with clinical phenotype and etiology also account for some of the variability in response. Recently, Horn *et al.* conducted a DBS sweet-spot mapping method, which demonstrated that for cervical dystonia patients, the most optimal stimulation result was achieved by modulating the striatopallidofugal axis of the basal ganglia [47^▪▪^]. It seems that optimal stimulation sites differ in different phenotypes, as the best result in generalized dystonia were achieved by stimulation of the pallidosubthalamic bundles. This suggests a short separation of optimal stimulation networks, with a common path back to thalamocortical level [47^▪▪^].

Over recent years, DBS devices able to record local field potentials from the macroelectrodes have become clinically available. Previous studies have shown evidence that theta oscillations in the GPi correlate with dystonic symptom severity and could serve as a physiomarker for dystonia [48]. DBS leads to a suppression of these theta oscillations [49]. Future directions for DBS are aimed at adaptive closed-loop systems. A recent proof-of-principle study of adaptive DBS (aDBS) in a single cervical dystonia patient showed that corticosubthalamic theta activity reduced with STN DBS [50^▪^].

For some patients, the dystonic symptoms are not sufficiently controlled by DBS alone [51,52]. Continuing prior/additional therapies can be necessary. A small size long-term management study in cervical dystonia patients showed optimal symptom control with at least one form of adjuvant therapy (BoNT and/or oral pharmacotherapeutics) [53]. Continuing adjuvant therapies should be considered in those patients that have not yet achieved satisfactory improvement of symptoms by solitary DBS treatment.

## PATIENT JOURNEY MAP

Recognizing the importance of holism regarding specific dystonia treatment, Benson *et al.* designed the first patient journey map aimed at cervical dystonia. This map provides a holistic view from prediagnosis to long-term treatment from the experience of the patient with idiopathic cervical dystonia [54^▪^]. On the map, five key stages are identified ranging from symptom onset to living with cervical dystonia with both the medical and emotional experience outlined. Development and implementation of these kinds of tools is important as involvement of patients in their own process makes treatment better tailored to their needs and, therefore, more relevant and of higher quality [54^▪^].

## ACCESS TO CARE FOR DYSTONIA PATIENTS

For patients, access to healthcare providers with expertise in dystonia can be challenging, with many countries still reporting difficult access to diagnostic tools such as genetic and neurophysiological testing, and treatment options including botulinum toxin and DBS [55^▪^]. This was even more complicated during the coronavirus disease 2019 (COVID-19) pandemic [56]. Nonetheless, the pandemic served as a catalyst in the use of telemedicine options, leading to a fundamental change in remote communication between healthcare providers and patients. Several studies have been conducted over recent years evaluating the application of telemedicine in dystonia. Its application has been broad, ranging from the programming phase in DBS to monitoring of the effect of BoNT [57,58]. Telemedicine seems to be an effective tool in the clinic and can help improve access to healthcare providers. It can be especially valuable to patients suffering from rare diseases such as dystonia, for whom care is often located at large tertiary centers that require traveling large distances.

## CONCLUSION

To conclude, there have been significant developments in the field of diagnosis and treatment of dystonia. Continuing efforts are being made to further develop recommendations and algorithms for dystonia subtypes to refine diagnosis and to assist in navigating the array of available diagnostic tools. There has been a growing interest over recent years in both motor and NMS associated with dystonia and their negative impact on quality of life. Future research is required to determine the effect of most commonly used treatment options (botulinum toxin, DBS) on the NMS, and on the exploration of treatment options specifically focused on these symptoms. Research focused on DBS is advancing in its search for electrophysiological biomarkers for dystonia. A line on the horizon in DBS for dystonia is the use of adaptive DBS systems.

## Acknowledgements

*None.*

### Financial support and sponsorship

*M.E. participated in a training program sponsored by Medtronic. M.A.J.T. reported grants from the Netherlands Organisation for Health Research and Development ZonMW Topsubsidie (91218013), the European Fund for Regional Development from the European Union (01492947) and the province of Friesland, from Stichting Wetenschapsfonds Dystonie Vereniging, and unrestricted grants from Actelion and Merz, AbbVie, Ipsen. L.M.C. reported grants from Merz and Stichting Wetenschapsfonds Dystonie.*

### Conflicts of interest

*There are no conflicts of interest.*

## REFERENCES AND RECOMMENDED READING

Papers of particular interest, published within the annual period of review, have been highlighted as:

▪ of special interest
▪▪ of outstanding interest
